# Postprandial hepatic stiffness changes on magnetic resonance elastography in healthy volunteers

**DOI:** 10.1038/s41598-021-99243-7

**Published:** 2021-10-05

**Authors:** Marzanna Obrzut, Vitaliy Atamaniuk, Jun Chen, Bogdan Obrzut, Richard L. Ehman, Marian Cholewa, Agnieszka Palusińska, Krzysztof Gutkowski

**Affiliations:** 1 Institute of Health Sciences, Medical College, University of Rzeszow, Rzeszow, Poland; 2 Department of Biophysics, College of Natural Sciences, Institute of Physics, University of Rzeszow, aleja Tadeusza Rejtana 16C, 35-959 Rzeszow, Poland; 3 Department of Radiology, Mayo Clinic, Rochester, MN USA; 4 Department of Obstetrics and Gynecology, Institute of Medical Sciences, Medical College, University of Rzeszow, Rzeszow, Poland; 5 Department of Internal Medicine and Nephrodiabetology, Medical University of Lodz, Lodz, Poland; 6 Institute of Medical Sciences, Medical College, University of Rzeszow, Rzeszow, Poland

**Keywords:** Liver cirrhosis, Liver fibrosis, Magnetic resonance imaging

## Abstract

Magnetic resonance elastography (MRE) is a reliable noninvasive method for assessment of hepatic stiffness. Liver stiffness is known to be affected by elevated postprandial portal blood flow in patients with chronic liver disease. The goal of this study was to determine whether food intake affects liver stiffness in the absence of known liver disease. We evaluated 100 volunteers (35 men and 65 women) who met inclusion criteria. The subjects had two MRE examinations, first while fasting and then 30 min after a test meal. Fourteen subjects also had two additional MRE exams 1 h 30 min and 2 h 30 min after the meal. Liver stiffness was measured by placing the largest possible polygon ROIs on the four widest liver slices and calculated as a mean of stiffness values from each slice. The correlation of liver stiffness values before and after the meal was assessed using a paired t-test. To evaluate the relationship between the change in postprandial liver stiffness and fasting liver stiffness values, linear regression was performed. The liver stiffness values in the fasting state ranged from 1.84 to 2.82 kPa, with a mean of 2.30 ± 0.23 kPa (95% CI 2.25–2.34). At 30 min after the meal, liver stiffness values ranged from 2.12 to 3.50 kPa, with a mean of 2.70 ± 0.28 kPa (95% CI 2.64–2.75), demonstrating a systematic postprandial increase by 0.40 ± 0.23 kPa (17.7 ± 3.5%). Meal intake significantly increases liver stiffness in healthy individuals, which persists for at least 2 h 30 min. Patients should fast for 3–4 h before MRE examinations to avoid fibrosis overstaging due to postprandial liver stiffness augmentation.

## Introduction

Chronic liver diseases are an important health problem worldwide. Regardless of the root cause (e.g., viral infection, alcohol abuse, and non-alcoholic fatty liver disease [NAFLD] in autoimmune diseases and genetically determined metabolic disorders), they often lead to liver fibrosis^1^. Hepatic fibrosis is a dynamic process that can be reversed if detected early and treated appropriately^2–4^. Delayed diagnosis and inadequate treatment of liver fibrosis can lead to liver cirrhosis with subsequent complications^5^. Liver biopsy with histopathologic analysis has long been regarded as the gold standard for the diagnosis of liver fibrosis^6^. However, this procedure is invasive, expensive, and affected by sampling error. Magnetic resonance elastography (MRE), has emerged as a non-invasive option for detecting and staging liver fibrosis with high diagnostic performance^6^. MRE uses magnetic resonance (MR) phase-contrast techniques to obtain images of propagating mechanical waves, generated in the liver by an external source of vibration. The images of waves are automatically processed by the MRI system to produce tissue stiffness maps called elastograms. Mean stiffness values are obtained from regions-of-interest (ROI) in the liver and used for interpretation.

Patient variables such as age, sex, obesity, and hepatic steatosis have been shown to have little or no effect on MRE-based measurements of liver stiffness^6^. Multiple studies have documented the relationship between MRE-assessed liver stiffness and the presence and stage of liver fibrosis^7–13^. MRE acquisition requires only a minute or less of imaging time, so the procedure can easily be included in a standard abdominal MRI protocol, enabling a comprehensive assessment that can also include rapid assessment of hepatic steatosis, and anatomic imaging to detect focal liver lesions and varices due to portal hypertension^5^. MRE is currently considered the noninvasive method with the highest performance for detecting and assessing liver fibrosis^5,6,14^. Normative values of liver stiffness in healthy people have been determined^15^.

It has been recommended that patients should fast for at least 4 h before MRE exams, in order to avoid transient postprandial liver stiffness augmentation that is known to occur in some patients^16^. This effect has been best documented in patients with chronic liver disease and could result in overstaging fibrosis^11,17,18^. However, less evidence is available about the magnitude of this effect in patients without a history of chronic liver disease.

The aim of the present study was to investigate whether food intake affects liver stiffness measurements in patients without clinical evidence of liver disease.

## Participants and methods

The eligibility criteria included: no history of liver disease; negative family history of chronic liver disease; normal aspartate transaminase (ASPAT 17–59 U/l for male and 14–36 U/l for female), alanine transaminase (ALAT < 50 U/l for male and < 35 U/l for female), lipase (23–300 U/l), and liver iron (49–181 µg/dL for male and 37–170 µg/dL for female) levels, no drug use, normal diet and alcohol consumption < 30 g/day. Exclusion criteria included: absolute contraindications for MR imaging, abnormal serum lipase, liver enzymes or liver iron, current or history of liver disease.

The research was carried out in the Magnetic Resonance Laboratory of the Center for Medical and Natural Sciences Research and Innovation of Rzeszow University, Poland, from December 2018 until July 2019.

### Study protocol

All participants were examined on a 1.5-T whole-body unit MR OPTIMA MR360 Advance (GE Healthcare, Milwaukee, WI, USA) with software version SV23. The device was equipped with a gradient system having a maximum amplitude of 33 mT/m and a slew rate of 120 mT/m/ms.

For liver MRI and MRE examinations, all participants underwent the scan in the feet-first supine position; an 8-channel torso phased array coil was placed over the patient’s abdomen. A drum-like passive driver was secured on the right side of the chest wall with its center at the xyphoid process. A 29-foot long polyvinyl chloride tube (3/4-in. diameter) connected the passive driver to an active driver, which was the source of vibration and stationed in the equipment room.

MR Touch (GE Healthcare) with a modified 2dimensional gradient‑recalled echo‑based pulse sequence and a 60-Hz frequency was used for the MRE examination. The parameters were as follows: field of view = 40 cm, repetition time = 33.3 ms, echo time = 20.5 ms, acquisition matrix = 224 × 64, recon matrix size = 256 × 256, slice thickness = 10 mm, flip angle = 30°; number of time offset segments = 4, time of acquisition = 1 min 8 s.

Nine slices were obtained through the liver in 3 or 4 breath-holds, with the participants asked to hold their breath at the end of expiration. Scanner console-generated elastograms were transferred offline for further analysis and measurements.

All participants underwent MRE examinations of the liver after fasting for at least 4 h, and then were provided a meal comprising two bananas, a serving of high-calorie yogurt, and a protein bar. The overall calorific value of the meal was 1000 kcal. The MRE examinations of the liver were repeated 30 min after the participants finished the meal. Additionally, for the group of 14 randomly selected participants, the liver stiffness assessment was also carried out at 1 h 30 min and 2 h 30 min after the meal.

To measure liver fat concentration, the IDEAL-IQ technique (GE Healthcare, Waukesha, WI, USA), with a gradient-echo multi-echo MR sequence, was used. The imaging parameters were as follows: FOV = 39 cm, TR = 12.4 ms, acquisition matrix = 160 × 160, slice thickness = 10 mm, slice number = 28. The scanner automatically generated fat-fraction images by collecting data at six echo times within a single breath-hold of 17 s.

### Image analysis

Liver stiffness was measured by placing 1 or 2 irregular polygon ROIs of various size and shape on the 4 widest liver slices in the right hepatic lobe on the elastograms. ROIs were drawn to encompass as much of the liver parenchyma as possible (mean size of the ROI in the liver was 5 to 7 cm^2^) while staying at least 2 mm within the outer liver capsule. In addition, areas of wave interference, large vessels, artifacts, and the central biliary tree were avoided when drawing the ROIs.

Liver stiffness was calculated as the mean of the mean liver stiffness values obtained from each of the 4 slices, weighted by the ROI area.

### Statistical analysis

Liver stiffness values are presented as mean ± SD. In our study, the liver stiffness in healthy volunteers was found to be relatively normal distributed in a narrow range. To assess the change of liver stiffness in the fasting state with liver stiffness after the test meal, a paired *t*-test was performed. Analysis of variance was used to assess changes in liver stiffness in the participants who underwent MRE/MRI at four time-points after the meal. Linear regression was performed to evaluate the relationship between the liver stiffness change and fasting liver stiffness values. Multivariate analysis was used to assess the correlation of the observed change in the liver stiffness with selected demographic, clinical, and laboratory parameters. Statistical significance was assumed at a *P* value of less than 0.05, and Bonferroni correction was applied for 4 time-points comparison. JMP Pro 14 (SAS Institute Inc., Cary, NC, USA) software was used to perform the statistical analysis.

### Ethics

The study protocol was approved by the Medical Department of the Rzeszow University Ethics Committee (Resolution No 10/01/2019) and conformed to the guidelines of the 1975 Declaration of Helsinki (6th revision, 2008). After receiving an explanation of the study, each participant provided written informed consent to enroll in the study.

## Results

Among the 115 participants, 15 were excluded because of abnormal (elevated) values of ASPAT and/or ALAT. The remaining 100 were considered eligible and were enrolled in the study. All MRE exams were successfully completed. None of the participants reported any discomfort during the procedure.

Of the 100 participants included in the study, 35 (35%) were men and 65 (65%) were women; mean age was 22.9 years (range 20–32 years). Mean (SD) body mass index (BMI) value was 21.33 ± 0.22 kg/m^2^ (95% confidence interval [CI], 20.89–21.76, range 16.85–25.76 kg/m^2^).

The liver stiffness value in the fasting state in the healthy participants ranged from 1.84 to 2.82 kPa, with a mean of 2.30 ± 0.23 kPa (95% CI 2.25–2.34). Mean liver stiffness for women was 2.31 ± 0.23 kPa (95% CI 2.26–2.37, range 1.84–2.82 kPa). For men, this parameter was 2.27 ± 0.23 kPa (95% CI 2.19–2.35, range 1.92–2.79 kPa). There was no correlation between liver stiffness and sex (*P* = 0.19).

The liver stiffness value after the meal in healthy participants ranged from 2.12 to 3.50 kPa, with a mean of 2.70 ± 0.28 kPa (95% CI 2.64–2.75), indicating an increase by 0.40 ± 0.23 kPa (95% CI 0.35–0.44, range 0.03–1.22) or 17.7 ± 10.7% (95% CI 15.57–19.83, range 1.06–61%).

Mean (SD) liver stiffness after the meal for women was 2.71 ± 0.28 kPa (95% CI 2.65–2.78, range 2.18–3.50 kPa). For men, this parameter was 2.67 ± 0.29 kPa (95% CI 2.57–2.76, range 2.12–3.31 kPa). Liver stiffness after the meal did not correlate with sex (*P* = 0.56; Table 1).

**Table 1:** Liver stiffness before and after the meal.

Liver stiffness, kPa		Mean value ± SD (95% CI)	Range	t-test
				Liver stiffness and sex
Before meal	Whole group	2.30 ± 0.23 (2.25–2.34)	1.84–2.82	P = 0.19
	Men	2.27 ± 0.23 (2.19–2.35)	1.92–2.79	
	Women	2.31 ± 0.23 (2.26–2.37)	1.84–2.82	
After meal	Whole group	2.70 ± 0.28 (2.64–2.75)	2.12–3.50	P = 0.56
	Men	2.67 ± 0.29 (2.57–2.76)	2.12–3.31	
	Women	2.71 ± 0.28 (2.65–2.78)	2.18–3.50	
Liver fat fraction, %	Whole group	2.51 ± 0.75 (1.40–4.90)	1.40–4.90	Liver fat fraction and sex
	Men	2.69 ± 0.88 (1.70–4.90)	1.70–4.90	P = 0.19
	Women	2.41 ± 0.66 (1.40–4.87)	1.40–4.87	
Liver stiffness change	kPa	0.4 ± 0.23 (0.35–0.44)	0.03–1.22	Linear regression test
				Liver stiffness change and liver stiffness before meal
	%	17.7 ± 10.7 (15.57–19.83)	1.06–61.0	P = 0.01

The liver fat fraction ranged from 1.40% to 4.90% (mean value was 2.51 ± 0.75% (95% CI 2.36–2.66%). For men, this parameter was 2.69 ± 0.88% (95% CI 2.39–2.99%), range 1.70–4.90%. For women, this parameter was 2.41 ± 0.66% (95% CI 2.25–2.58%), range 1.40–4.87%. The liver fat fraction did not correlate with sex (*P* = 0.19; Table 1).

**Figure 1:**
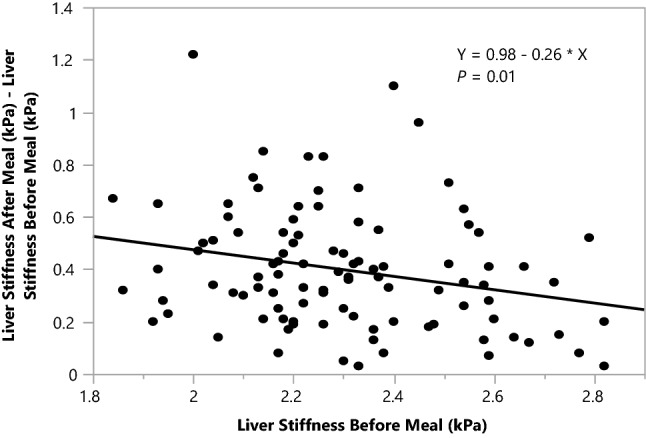
Correlation between liver stiffness difference and liver stiffness before meal. The increase in the liver stiffness before and after the meal was negatively correlated with the liver stiffness values before the meal (*P* = 0.01).

Participants with lower fasting liver stiffness had a significantly larger postprandial stiffness increase than higher fasting liver stiffness (*P* = 0.01; Fig. 1).

Multivariate analysis revealed that among the selected demographic, clinical, and laboratory parameters, only “fat layer thickness” (*P* = 0.03) and “fasting liver stiffness (*P* = 0.04) were significantly correlated with the increase in liver stiffness after the meal (Table 2).

**Table 2:** The multivariate analysis of correlation.

Source	P value
Fat layer thickness (mm)	0.028*
Fasting liver stiffness (kPa)	0.035*
Age	0.081
BMI, kg/m^2^	0.231
Fat fraction liver, %	0.251
HGB, g/dL	0.332
ASPAT, U/I	0.343
ALAT, U/I	0.574
RBC, × 10^6^/µL	0.636
Ferritin, ng/ml	0.686
PLT, × 10^3^/µL	0.698
Lipase, U/I	0.728
WBC, × 10^3^/µL	0.733
HCT, %	0.744
Sex	0.757
Iron, µg/dL	0.988
R2* liver	0.995

Multivariate analysis showing that only “fat layer thickness” and fasting liver stiffness were significantly correlated with the increase liver stiffness increase before and after the meal.

*P < 0.05.

In the 14 participants who had liver stiffness measurements before the meal and at 30 min, 1 h 30 min, and 2 h 30 min after the meal, the liver stiffness value reached a maximum at 30 min and then slowly decreased during the observation time window. To protect from Type I Error, we have added the post hoc Bonferroni correction for the multiple time-points comparison. There were four timepoints, therefore the p values for the statistically significance level is modified as 0.05/4 = 0.0125.

**Figure 2:**
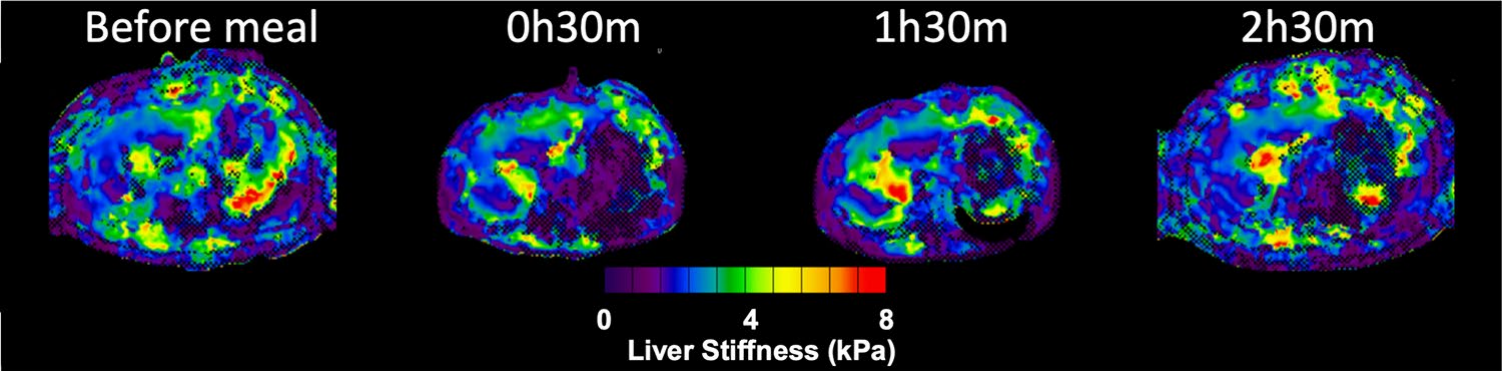
Liver stiffness maps at different time points. Example of liver stiffness maps generated by a scanner before and at 30 min, 1 h 30 min, and 2 h 30 min after the meal.

Even at 2 h 30 min after the meal, the liver stiffness value was greater than fasting liver stiffness (Table 3, Fig. 2).

**Table 3:** Liver stiffness at different time-points in a group of 14 randomly selected participants.

Liver stiffness at different time-points, kPa			
	Mean value	SD	95% CIs (lower, upper)
Before meal	2.25	0.26	2.10–2.41
30 min after meal	2.71	0.35	2.56–2.86
1 h 30 min after meal	2.52	0.27	2.37–2.67
2 h 30 min after meal	2.41	0.23	2.26–2.56

**Figure 3:**
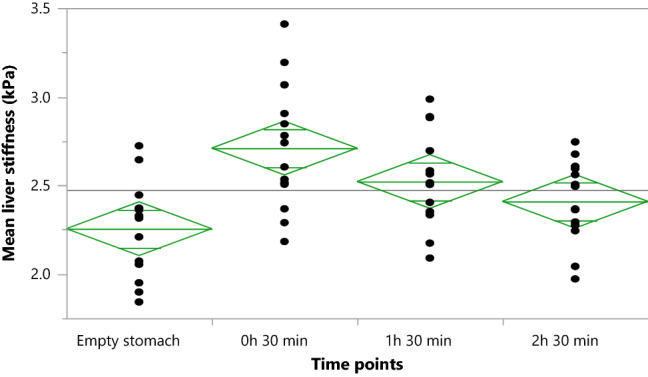
Liver stiffness values at different time points. Liver stiffness before and at 30 min, 1 h 30 min, and 2 h 30 min after the meal.

The effect of the meal on liver stiffness was statistically significant (repeated measures analysis of variance, *P* < 0.01; Fig. 3).

## Discussion

The presented research is the largest prospective study to evaluate postprandial MRE-based stiffness liver changes in patients with no previous history of chronic liver disease. We observed a postprandial increase in liver stiffness by 0.40 ± 0.23 kPa (17.7 ± 10.7%0. In the MRE literature, Yin et al.^17^ reported a postprandial increase in liver stiffness after meal by 0.16 ± 0.20 kPa (8.1% ± 10.3%). The postprandial increase in liver stiffness in our study was higher, which may be attributed to differences between the patient populations and the meal used in the experiment.

The presented results are significant for clinical practice and reinforce the validity of the recommended fasting period before MRE examinations. While the mean difference of 17.7% is within the range of test–retest variability (19%) documented in the QIBA consensus profile for MRE^16^, the observed effect could result in incorrect staging of liver fibrosis even in patients without a history of chronic liver disease. The threshold for diagnosing stage 1 fibrosis with MRE has been estimated to be in the range of 3.0 kPa in many published studies. Using that threshold, 15% of the subjects in this study would have incorrectly been diagnosed with stage 1 fibrosis on the basis of non-fasting measurements, whereas all would have been classified as no fibrosis in the fasting exams.

Liver stiffness measurements (LSM) are considered to be influenced by 2 factors: a static factor reflecting the structural properties of tissues, including changes in the extracellular matrix occurring during the course of inflammatory and fibrotic processes^8,19,20^, and a dynamic factor reflecting perfusion pressure^21^. Changes in hepatic artery flow parameters potentially affect the LSM: after a meal, the flow rate decreases while the hepatic artery resistance index increases^22^. According to Ohm's law, an increase in the portal vein flow after a meal would increase portal pressure, provided the resistance of intrahepatic vessels remains constant. Autoregulation of blood flow may depend on intrahepatic pressure at the level of the vascular bed, which maintains constant pressure parameters in the portal bed^23^. Currently, the researchers are inclined toward the thesis that changes in intrahepatic pressure caused by increased blood flow in the portal vein and postprandial hyperemia of the liver lead to changes in sinusoidal pressure, thereby preventing overpressure on the portal system in healthy people^17^. If autoregulation is impaired, an increased portal vein flow will increase venous pressure and therefore also tissue stiffness^17^. Indeed, researchers describe as much as a 30% to 40% increase in the portal blood flow and portal blood velocity and hepatic venous pressure gradient after a meal in patients with cirrhosis^24^.

In our study, the highest average liver stiffness value was recorded 30 min after a meal. Liver stiffness then gradually decreased, but even at 2 h 30 min after the meal, the liver stiffness was greater than that measured with an empty stomach: 2.41 vs. 2.25 kPa. This finding is consistent with reports by others who demonstrated a decrease in liver stiffness compared with baseline values after more than 3 h after consuming a meal^25^.

In addition, we observed that patients with lower liver stiffness levels on an empty stomach had greater increases in postprandial liver stiffness. Identical observations were reported by Petzold et al.^26^. It remains to be determined whether a similar phenomenon occurs in sick people.

Our study revealed no correlation between liver stiffness and age, sex, or selected laboratory parameters. Similarly to Yin et al.^17^, we showed no correlation between hepatic stiffness and BMI, in contrast to Petzold et al.^26^, who reported a significant positive correlation between postprandial LSM and BMI, using ultrasound based elastography.

One limitation of the presented research is the narrow age range of the study group. Therefore it was not possible to investigate the postprandial liver stiffness changes with reference to different age subgroups. Further studies should involve participants within a wider age range to analyze such potential differences. It would also be very interesting to extend the investigations on other populations as a multicenter study to confirm the universalism of presented results.

## Conclusion

Meal intake causes significant increase of liver stiffness in healthy individuals. Postprandial liver stiffness changes persist for more than 2 h 30 min. Patients should fast for 3–4 h before MRE examinations to avoid fibrosis overstaging due to postprandial liver stiffness augmentation.

## Data Availability

The data that support the findings of this study are available from the corresponding author upon reasonable request.
